# Effects of Genetic polymorphism in susceptibility to schistosomiasis infection and adverse treatment outcomes

**DOI:** 10.4314/ahs.v25i3.2

**Published:** 2025-09

**Authors:** Akingbolabo Daniel Ogunlakin, Oluwafemi Adeleke Ojo, Oluwaseun Abigael Ogunlakin, Ajibola David Adelakun, Ayobami Tosin Adegbenro, Favour Inijesunimi Olagookun, Sophie Adedamola Adeyeye, Mubo Adeola Sonibare

**Affiliations:** 1 Bowen University SDG 03 (Good Health and Wellbeing Research Cluster), Iwo, Nigeria; 2 Phytomedicine, Molecular Toxicology, and Computational Biochemistry Research Laboratory (PMTCB-RL), Biochemistry Programme, Bowen University, Iwo, 232101, Nigeria; 3 Agricultural sciences programme, Bowen University, Iwo, 232101, Nigeria; 4 Department of Pharmaceutical Sciences, University of Arizona, Arizona, USA; 5 Department of Biochemistry and Molecular Biology, Obafemi Awolowo University, Ile-Ife, Nigeria; 6 Department of Plant Science, Olabisi Onabanjo University, Ago-Iwoye, Ogun State, Nigeria; 7 Department of Pharmacognosy, University of Ibadan, Ibadan. Nigeria

**Keywords:** Tropical disease, Schistosomiasis, Genetic polymorphism, Primary locus, Chromosome

## Abstract

Schistosomiasis is a serious tropical disease that is undiagnosed (Neglected tropical disease -NTD). The only disease with a more detrimental socio-economic impact than its causative agent is malaria. Over 250 million individuals are presently afflicted with Schistosomiasis haematobium, and an additional 779 million people are at risk of infection in regions where the disease is endemic. Africa is home to many incidences, which account for roughly 90% of infections. Every year in underdeveloped nations, schistosomiasis claims the lives of 11,700 people and disables over 3.3 million others. The parasite is still dangerous everywhere in the world, even in non-endemic places. This review delves deeply into genetic polymorphism and its different forms. It also addresses genetic polymorphism to susceptibility. It has been found that environmental factors are among the many variables that influence susceptibility to schistosomiasis. Schistosomiasis susceptibility is influenced by age, gender, and even pathological infections. The primary locus SM1, situated on chromosome 5q31-q33, regulates the extent of S. mansoni and S. haematobium infection by including multiple genes associated with the Th2 immune response. The chromosome 5q31–q33 Th2 cluster gene, which regulates the synthesis of interleukin 13, interleukin 4, and interleukin 5, is primarily linked to an increased risk of developing schistosomiasis.

## Introduction

Schistosomiasis is the world's fourth most common parasite disease, affecting around 200 million people^1^-^3^. Numerous parasitic worm species in the genus Schistosoma are responsible for schistosomiasis. Schistosoma haematobium, Schistosoma japonicum, and Schistosoma mansoni are the three primary species that infect people.

These organisms cause several types of illness that impact the intestines or the urinary tract^4^,^5^. Disrupting the parasite, host, and carrier life cycles has been the primary goal of control efforts^6^,^7^. Nevertheless, most parasite eggs are only released into the environment by a tiny percentage of infected people, resulting in an unequal distribution of the parasites^8^-^10^. Hereditary and environmental factors may contribute to these unequal distributions^11^,^12^. Improved control methods might result from a deeper comprehension of the genetic variables influencing the severity of infections and diseases^13^-^15^. Thus, comprehending the genetics of elevated worm load and susceptibility to schistosomiasis could prove beneficial in developing therapeutic interventions, including vaccination^15^,^16^.

Despite efforts to treat it, schistosomiasis is still a serious public health concern with unfavourable results. The main treatment, praziquantel, works well but has drawbacks^17^. Pregnancy-related schistosomiasis is associated with intrauterine growth retardation, low birth weight, and maternal anaemia. According to a Nigerian study, pregnant women with schistosomiasis experienced greater rates of anaemia and considerably lower birth weights than women without the infection^18^. Poor maternal and newborn health outcomes may result from the immune system's reaction to the parasite^19^. Although praziquantel works well against all of the main Schistosoma species, the host's immune response may affect treatment effectiveness^20^. There is little indication of parasite resistance, and in certain situations, additional treatments are required^21^. Another major issue is the paucity of safety trial data regarding the use of praziquantel in pregnant women and children under the age of four^22^. Limited evidence suggests that praziquantel-resistant parasites exist, especially in highly infected populations^23^. Confirming the effectiveness of treatment requires follow-up exams, particularly in regions with high rates of reinfection^24^. To address these issues, a multimodal strategy is needed, one that includes ongoing research into alternative medicines, greater follow-up care, and enhanced treatment protocols.

Seventy eight nations have endemic populations of the six schistosome species, but only three majorly affect people worldwide, namely Schistosoma japonicum, Schistosoma haematobium, and Schistosoma mansoni^25^. S. haematobium, which causes urogenital schistosomiasis, is closely associated with viral illnesses including Human Immunodeficiency Virus and HPV.^26^ Aula et al. 27 report that Schitosomia haematobium are distributed over a large geographic range, encompassing areas of Africa, Madagascar, the Indian Ocean Islands, and Egypt. S. haematobium infections in people are expected to out-number other schistosome species by over 112 million cases in sub-Saharan Africa. Several snail species act as intermediate hosts. S. haematobium was previously prevalent throughout Egypt, but it is currently only found in populations with a range of 0% to 13.9% as a result of national control activities^28^. Recent research shows that environmental and genetic factors might contribute to an individual's infection in an endemic area^29^,^30^. Particularly, intriguing is the presence of several genetic variables connected to immune function in the human genomic region 5q31-q33. Among these groups is the T-helper 2 (Th2) gene cluster, which regulates the synthesis of IL-4, IL-13, and IL-5 during immune responses^31^. Therefore, this review presents the effects of genetic polymorphism in susceptibility to schistosomiasis infection and adverse treatment outcomes.

## Method

Using the keywords “schistosomiasis”, “genetic polymorphism”, “adverse treatment outcomes”, “epidemiology” and “treatment of schistosomiasis”, and “role of genetic polymorphism”, a search was done in Google Scholar, Pubmed/Medline, and Scopus from the beginning of the databases to February 2024. The references of all papers were checked for cross-references that had not been found in the database search.

### Genetic polymorphism

**Figure 1:**
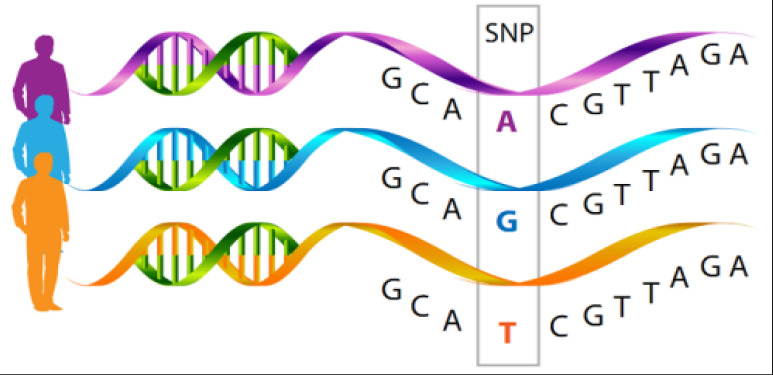
Genetic Sequencing indicating Single Nucleotide Polymorphism^35^

Polymorphism refers to the existence of multiple forms of genes. It demonstrates how genomics introduces new questions. Although humans are essentially identical, the sequencing of human genomes has revealed small differences between individuals known as single nucleotide polymorphisms, or SNPs^32^. DNA polymorphism has a wide range of variants, numerous united pairs, and frequent ordering. One of the most well-known types of genetic variants is genetic mutations^33^. Less than 1% of the population possesses a genetic mutation, which is classified as an order variant; more prevalent variants are called polymorphisms. Less than 1% of public hereditary variants are SNPs (SNPs)^34^. Genetic polymorphism generally comes in different types, such as Single Nucleotide polymorphism (SNP; Figure 1), Short tandem repeats (STRs), Variable number of tandem repeats 9VNTRS), Insertion and deletion polymorphism, Transposable elements (TE), and Copy number variations^34^.

### Role of Genetic Polymorphism in Susceptibility to Schistosomiasis

Schistosomiasis, the fourth most common parasite disease in the world, affects about 200 million people^36^. According to Faust et al.,^37^ the primary objective of control measures has been to disrupt the parasite, carrier, and host response life cycles. The geographical distribution of the parasites is greatly skewed since only a small fraction of infected people release the majority of the parasite eggs into the environment. Understanding the genetic mechanisms behind infection intensity and disease could lead to improved control techniques^38^. These skewed distributions may have been caused by both inherited and environmental causes^39^. The major is the potential impact of genetic factors on parasite burden. The primary locus SM1, located on chromosome 5q31–q33, contains multiple genes associated with the Th2 immune response and regulates the degree of infection with S. mansoni and S. haematobium. Hepatic and portal vein fibrosis are two conditions that are commonly associated with schistosomiasis. Additionally, there is a connection between the severity of fibrosis and the SM2 quantitative trait locus (QTL) gene located on chromosome 6.15

S. mansoni and S. haematobium, digenic trematodes of the genus Schistosoma, are responsible for the majority of human infections caused by schistosomiasis^40^. Fresh water snails are infected by adult parasites that are situated in veins close to the bladder and intestines, and whose eggs are released into the urine or feces^13^. When people enter the water, the number of parasites increases in the intermediate host snail, which then releases the parasites in stages that are infectious to humans and can pierce the skin. Schistosomiasis not only induces liver and bladder fibrosis but can also lead to colon cancer in the long run. Individuals with schistosomiasis experience acute, severe, and chronic morbidity^41^. Although exposure to Schistosome cercariae-contaminated water is the main cause of schistosomiasis, the degree of infection varies greatly across people under comparable circumstances, and instances of schistosomiasis typically run in families. Some of these variations are attributed to the genetic coding of the human immune system response^15^.

### Immune Response to Schistosoma Infections

**Figure 2:**
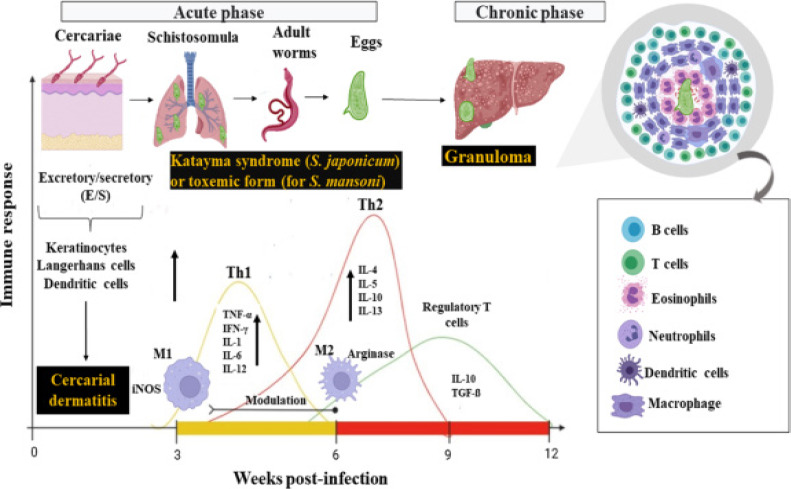
Different immunological response profiles when infected with S. japonicum and S. mansoni^49^

According to Costain et al.^42^ Th1 immune responses, as shown in Figure 2, are mainly responsible for combating schistosoma larvae that enter and move, as well as adult strains. Innate cells get activated, excretory Schistosoma antigens damage host barrier cells, and pro-inflammatory cytokines such as IFNG, IL-7, IL-16, TNF, and IL-1β are generated along with alarmins^43^. After infection, 6-7 weeks later, schistosoma eggs are discharged into the liver, colon, or bladder, which causes Th2 cells to multiply^44^. Additionally, schistosoma egg antigens bind to antigen-presenting cell receptors with efficiency, preventing the production of IL-12 and subsequent Th1 responses^45^. Pre-patent IL-4 is generated by CD4 T cells in response to single-sex schistosome infection, which can also initiate the Th2 response in the absence of egg deposition. Long-term infections diminish Th2 responses specific to schistosomes, which are associated with the development of regulatory cells that produce transforming growth factor beta, or “TGFβ,” and IL-10^45^. This enables the parasite to remain in the host and reduces harm to host tissue. It also controls the host's immune system's reaction to other unrelated antigens, such as immunizations, self-antigens, and allergies^15^,^46^.

A substantial amount of antigenicity and polarizing granulomatous Th2 responses are typically induced by schistosome egg secretions^47^. Granulomas develop around parasite eggs to shield tissue cells from the eggs being ingested by the body. On the other hand, fibrosis is set on by persistent host CD4 Th2 cell-mediated reactions to the parasite eggs. Human periportal fibrosis is associated with the Th2 cytokine IL-13. Elevated IL-17 levels and IFNG control of hepatic inflammatory alterations in S. mansoni-infected mice are connected, beyond the Th2 cytokine responses. T regulatory cells are associated with the lower prevalence of human schistosomiasis; yet anomalies in ultrasonography textural features are linked to CD4 T helper cells, which generate IL-17^15^,^48^.

### Phenotypes

Pathology-related and infection-associated phenotypes are the two categories of phenotypes that have been the subject of studies on the human genetics of susceptibility to schistosomiasis^50^. While worm load estimates and egg counts are frequently associated with infections, total IgE can also be used as a measure of the immune system's response^51^,^52^. The method of urine filtration and the Kato Katz (KK) procedure are used to determine the egg counts for S. mansoni and S. haematobium, appropriately. Worm loads are estimated based on the amount of circulatory cathodic antigen (CCA) that mature worms release in urine^15^,^53^.

Egg loads for schistosomes rise until adolescence and then fall, exhibiting a similarly high skew in the age distribution^54^. A Brazilian study^54^ found that egg burdens were nearly ten times higher in children under the age of 19 than in adults. Since the development of schistosome immunity takes time, children may be more susceptible to infections and more frequently with them. IgE responses that are protective against larvae can be triggered by the antigens expelled by dying worms interacting with antigens on the larvae^55^. It requires exposure to enough dying worms over a prolonged period (5–16 years) for children to acquire an IgE response to the larvae. High levels of IgG4 have been linked to susceptibility, although larger levels of anti-parasite have already been linked to resistance^15^, ^46^.

### Schistosome linked with hepatic and periportal fibrosis and portal hypertension

According to Santana et al.^56^ and Hashim and Berzigotti^57^, fibrotic lesions can form around egg granulomas in various tissues, and schistosomes can cause a wide range of symptoms. However, hepatic fibrosis (HF) and periportal fibrosis (PPF) are the two main markers of S. mansoni and S. japonicum pathology. PPF and HF scoring measures, as recommended by the WHO, are used in genetic investigation. The extracellular matrix that surrounds schistosome eggs is what causes HF and PPF^58^. The hepatic portal vein may experience portal hypertension (PH) because of internal bleeding, superinfections, heart failure, or renal failure and some PH patients pass away from the illness^15^,^59^-^61^. Though S. haematobium and S. mansoni are linked to bladder cancer and hepatocellular carcinoma, respectively, most of the genetic research on schistosomiasis has focused on HF and PF^62^. Fibrosis can be identified by ultrasonic scanning, despite concerns about the method's accuracy and consistency. There are currently efforts underway to generate more indicators and criteria for pathology evaluation, however, these lack adequate confirmation^15^.

### Heritability of schistosomiasis infection risk

Noticeable risk requires a large heritability or the proportion of risk attributable to genetic differences. The estimates of schistosomiasis heritability are compiled in Table 1. In several studies, additional heritability (h2) values ranging from 23 to 31% have been employed to approximate comparable levels of genetic component variation-related variability in *S. mansoni* egg quantity^15^.

**Table 1:** Estimates of the genetic risk of schistosomiasis in various populations based on heritability^15^

Parasite	Phenotype	Country and District	Heritability estimates and methods eg *h2*
*S. mansoni*	Egg count	Brazil, Minas Gerais	23% (*h2*)
*S. mansoni*	Egg count	Brazil, Minas Gerais	27% (VC)
*S. mansoni*	Egg count	Brazil, Bahia	31% (VC)
*S. mansoni*	IgE	Brazil, Bahia	59% (VC)
*S. haematobium*	Egg count	Kenya, Coast	9% (*h2*)
*S. haematobium*	Bladder morbidity	Kenya, Coast	14% (*h2*)
S. *japonicum*	Infection	China, Jiangxi	58% (VC)
S. *japonicum*	Infection	China Sichuan	0% (VC)

h2 additive heritability, VC variance compounds

### Linkage studies on schistosomiasis

Segregation analysis was used in the first investigation of the biology of human schistosomiasis to investigate if the existence of a significant gene might explain the disease's incidence in familial pedigrees^63^. This study used parametric linkage analysis to demonstrate the existence of a significant gene that was subsequently named SM1 and mapped to a chromosome^15^. The first quantitative trait locus for any infectious disease discovered in humans was the SM1 quantitative trait locus (QTL) for *S. mansoni* fecal egg count on chromosome 5q31-q33.^64^ Table 2 presents Loci associated with *S. mansoni* infection discovered by image studies as reported by Mewamba et al^15^. One reason for the remarkable success of this investigation was the relatively large correlation value of the SM1 loci (65% of the variation after controlling for age, sex, and water contact). This stands in stark contrast to the relatively small shares of variance explained by the majority of GWAS locations^15^. The same team that carried out the initial study in Brazil followed up with research in Senegal and found a correlation at the SM1 gene. The relationship could only be proven by testing for effects at the SM1 locus because the effects were not as substantial^15^.

**Table 2:** Loci associated with *S. mansoni* infection discovered by image studies^15^

Phenotypes	Locus name	5′ Marker (Position)	3′ Marker (Position)	Candidate genes, Tested (Untested)
Egg count	SM1 5q31-q33	D5S642 (128Mb)	D5S412 (158Mb)	*IL-4, IL-5, IL-9, IL-13* *(IL-3, CXCL14, CD14*, *1IL-17β, IL12β)*
Egg count	1p21-q23	D1S236	D1S196	*(IL6R, CRP)*
		(95Mb)	(168Mb)	
Egg count	6p21-q21	D6S271	D6S283	*(VEGFA*, *IL-17A, IL-17F)*
		(43Mb)	(67Mb)	
Hepatic fibrosis	SM2 6q22-q23	D6S1009	D6S310	*CTGF* *IFNGR1, IL--22RA2*
		(137Mb)	(142Mb)	
Egg count	7q35-q36	D7S483	D7S550	*(TRB, NOS3, SHH)*
		(152Mb)	(156Mb)	

**Figure 3:**
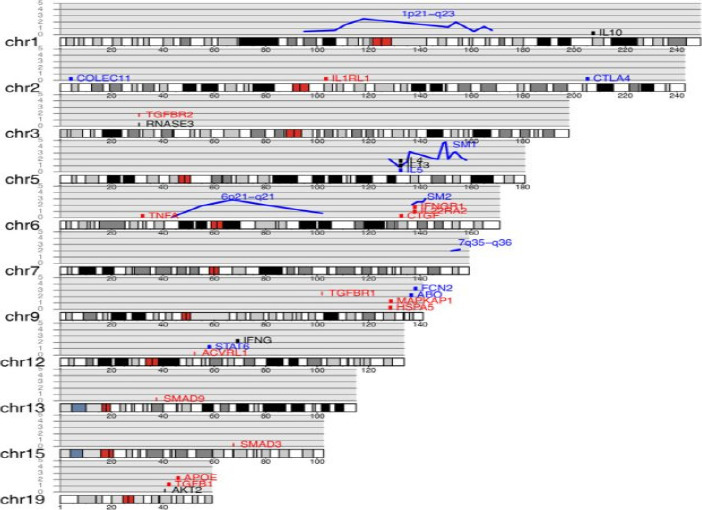
The human karyotype is used to map the genes and quantitative trait loci linked to schistosomiasis. With the reported −log p-value for association represented on the y-axis, blue lines denote quantitative trait locus (QTL). Genes implicated in pathology are indicated in red, genes harboring SNP linked to schistosomiasis infection are presented in blue, and genes involving both pathology and infection are shown in black. Genes are plotted vertically for clarity, with their y-axis position being arbitrary^15^

To further account for Schistosomiasis haematobium, a second experiment was conducted in four endemic villages in Ghana to ascertain the relationship between the IL-13 gene and the susceptibility to schistosomiasis infection. The ages of the experiment participants ranged from six to nineteen. IL-13-591A/G, IL-13-1055C/T, and IL-13-1258A/G were correlated with varying levels of Schistosoma haematobium contamination. Among the genes associated with immune function found in this context, of particular importance is the human genomic region 5q31-q33. The T-helper 2 (Th2) cluster, a group of genes implicated in this field, oversees producing IL-4, IL-13, and IL-5 during the immunological response. They concentrated on the single-nucleotide polymorphisms (SNPs) in the IL-13 promoter region, particularly IL-13-1055C/T, IL-13-591A/G, and IL-13-1258A/G^65^. Figure 3 presents the application of the human karyotype used to map the genes and quantitative trait loci linked to schistosomiasis^15^. In candidate gene investigations, SNP has been linked to schistosomiasis infection characteristics, as presented in Table 3^15^.

**Table 3:** In candidate gene investigations, SNP has been linked to schistosomiasis infection characteristics 15

SNP	Gene	Phenotype	Parasite
rs2430561	IFNG	T2R	Sm
rs3024495	IL10	FEC	Sm
rs1800896	IL10	IgE	Sm
rs1800871	IL10	IgE	Sm
rs1800872	IL10	IgE	Sm
IL 10(-1082/-819/-592)	IL10	UEC	Sh
rs20541	IL13	FEC	Sm
rs2066960	IL13	FEC	Sm
rs7719175	IL13	UEC	Sh
rs2069743	IL13	UEC	Sh
rs1800925	IL13	UEC, FEC, T2R	Sh, Sm
rs2243250	IL4	UEC, T2R	Sh
rs2079103	IL5	IF	Sj
rs2706399	IL5	IF	Sj
rs3024974	STAT6	UEC	Sh
rs324013	STAT6	UEC	Sh
rs733618	CTLA4	UEC	Sh
rs11571316	CTLA4	UEC	Sh
rs231775	CTLA4	UEC	Sh
rs3124952	FCN2	UEC	Sh
rs17514136	FCN2	UEC	Sh
rs7567833	COLECC11	UEC	Sh
COLEC11*TCCA	COLECC11	UEC	Sh
Blood group O	ABO	FEC, UEC	Sh, Sm
Rs746822072	RNASE3	UEC	Sm

### Age, gender and Schistosoma haematobium infection

Investigation demonstrated that pupils between the ages of 11 and 14 had the highest incidence of *S. haematobium* infection, while those between the ages of 15 and 19 had the lowest prevalence^66^-^68^. A corresponding pattern was observed in the geometric mean of *S. haematobium* infection intensities, which increased with age, peaked between the ages of 11 and 14, and then decreased with age. However, male students had a higher incidence of *S. hematobium*^31^. It was also discovered to what extent the 71 students who were evaluated had IL-13 genetic variations linked to *S. haematobium* infections, age, and gender. For each of the three IL-13 gene polymorphisms that were taken into consideration—IL-13-1055C/T (i.e., C/C, T/T, and C/T), IL 13-591A/G (i.e., A/A, G/G, and A/G), and IL-13-1258A/G (i.e., A/G, G/G, and A/G)—there was no statistically significant difference between the two age strata^31^.

Additional classification based on gene polymorphisms showed that pupils with the genotypes IL-13-1055C/C or IL-13-1055C/T, IL-13-591A/A or IL-13591A/G, and IL-13-1258A/A or IL-131258A/G had increased infection rates, particularly in the 11–14 age range. Genotypes with the IL-13-1055C, IL-13-591A, and IL-13-1258A alleles had an overall greater prevalence rate of *S. haematobium* infection. Furthermore, those who were homozygous for the IL-13-1055T (i.e., T/T), IL13-591G (i.e., G/G), and IL13-1258G (i.e., G/G) alleles had the lowest prevalence of S. haematobium infection^31^. In rural regions, the estimated prevalence of schistosomiasis was 22%. People with higher prevalence and severity of *S. haematobium* infection were found to carry the IL-13-1055C, IL-13-591A, and IL-13-1258A alleles. In addition, individuals with the IL-13–1055C, IL-13–1258A and IL-13–591A alleles in the 1q–14 age group showed a higher probability of S. haematobium infection. It is important to note that people who had the IL13-1258A, IL13-591A, and IL13-1055cA alleles also had increased STH prevalence^31^,^69^.

## Conclusion

Schistosomiasis is a serious tropical disease that is undiagnosed (NTD). Schistosomiasis susceptibility is influenced by age, gender, and even pathological infections. The primary locus SM1, situated on chromosome 5q31-q33, regulates the extent of *S. mansoni* and *S. haematobium* infection by including multiple genes associated with the Th2 immune response. The chromosome 5q31–q33 Th2 cluster gene, which regulates the synthesis of interleukin^13^, interleukin^4^, and interleukin^5^, is primarily linked to an increased risk of developing schistosomiasis.
